# The Impact of Simulated and Real Microgravity on Bone Cells and Mesenchymal Stem Cells

**DOI:** 10.1155/2014/928507

**Published:** 2014-07-10

**Authors:** Claudia Ulbrich, Markus Wehland, Jessica Pietsch, Ganna Aleshcheva, Petra Wise, Jack van Loon, Nils Magnusson, Manfred Infanger, Jirka Grosse, Christoph Eilles, Alamelu Sundaresan, Daniela Grimm

**Affiliations:** ^1^Department of Physiology, Membrane Physiology, University of Hohenheim, 70593 Stuttgart, Germany; ^2^Clinic for Plastic, Aesthetic and Hand Surgery, Otto-von-Guericke University, 39120 Magdeburg, Germany; ^3^Hematology/Oncology, Children's Hospital Los Angeles, University of Southern California, Los Angeles, CA 90027, USA; ^4^Department of Oral and Maxillofacial Surgery/Oral Pathology, VU University Medical Center Amsterdam, 1007 MB Amsterdam, The Netherlands; ^5^Department of Oral Cell Biology, Academic Centre for Dentistry Amsterdam (ACTA), University of Amsterdam and VU University Amsterdam, 1081 LA Amsterdam, The Netherlands; ^6^European Space Agency Technology Center, Gravity Lab (ESA-ESTEC-TEC-MMG), 2201 AZ Noordwijk, The Netherlands; ^7^Medical Research Laboratory, Institute of Clinical Medicine, Aarhus University, 8000 Aarhus C, Denmark; ^8^Department of Nuclear Medicine, University of Regensburg, 93052 Regensburg, Germany; ^9^Department of Biology, Texas Southern University, 3100 Cleburne, Houston, TX 77004, USA; ^10^Institute of Biomedicine, Pharmacology, Aarhus University, Wilhelm Meyers Allé 4, 8000 Aarhus C, Denmark

## Abstract

How microgravity affects the biology of human cells and the formation of 3D cell cultures in real and simulated microgravity (r- and s-*µg*) is currently a hot topic in biomedicine. In r- and s-*µg*, various cell types were found to form 3D structures. This review will focus on the current knowledge of tissue engineering in space and on Earth using systems such as the random positioning machine (RPM), the 2D-clinostat, or the NASA-developed rotating wall vessel bioreactor (RWV) to create tissue from bone, tumor, and mesenchymal stem cells. To understand the development of 3D structures, *in vitro* experiments using s-*µg* devices can provide valuable information about modulations in signal-transduction, cell adhesion, or extracellular matrix induced by altered gravity conditions. These systems also facilitate the analysis of the impact of growth factors, hormones, or drugs on these tissue-like constructs. Progress has been made in bone tissue engineering using the RWV, and multicellular tumor spheroids (MCTS), formed in both r- and s-*µg*, have been reported and were analyzed in depth. Currently, these MCTS are available for drug testing and proteomic investigations. This review provides an overview of the influence of *µg* on the aforementioned cells and an outlook for future perspectives in tissue engineering.

## 1. Introduction

It is well known, that microgravity influences different biological systems like bone and muscle as well as the heart and brain, and it enhances cancer risk [1]. During their stay at the MIR, astronauts and cosmonauts did show a distinct loss of bone mineral density in the lumbar spine, the pelvis, and the proximal femur [2], and the extent of bone loss varied up to 20% [3].

As it is not feasible to gather enough material from astronauts to do in-depth investigations, another device has been developed for the International Space Station (ISS), the mice drawer system (MDS), as a facility to study long-time influence of radiation on the biology and behavior of mice. Tavella et al., for example, report an altered bone turnover in different strains of mice which were kept on the ISS for 91 days. This resulted in bone loss due to increased bone resorption and a decreased bone deposition [4].

While the past biological, physiological, and medical research nearly exclusively focused on investigating the biochemical processes of living cells and organisms, more and more attention was paid to the biomechanical properties and mechanical environment of cells and tissues during the last decades. When culturing cells on Earth, they usually settle on the bottom of the culture flask, forming two-dimensional (2D) monolayers. A three-dimensional (3D) growth, more resembling the tissue environment found in living organisms, is prevented by the presence of the gravitational field. For a scaffold-free 3D tissue growth, it is therefore necessary to circumvent this problem by effectively eliminating the influence of the gravitational pull during cultivation. One of the byproducts of various space flight endeavors is the possibility to perform long-term near-weightlessness or microgravity (*μg*) experiments [5, 6]. In a *μg* environment, cells will not settle like on Earth. This provides an increased opportunity for freely floating cells to interact with each other and develop 3D structures [7].

## 2. Space Flights for Cell-Biological Experiments

Long-term orbital space flight experiments are, however, not trivial. Flight opportunities are very scarce and the costs of hardware development are high. Furthermore, science is not always a priority in space flight activities. Such preconditions are delaying the advancement of research in areas such as cell biology and tissue engineering disciplines, which could profit tremendously from more frequent research options in a real microgravity (r-*μg*) environment.

Some researchers recently pointed out that osteoblasts undergo a disintegration of their cytoskeleton, which may explain dramatic changes in size and shape of the cells and their surface specializations [47]. Also, other studies have been performed using the ISS or space shuttle flights to learn more about the behavior of bone cells in space [48], but flight opportunities are sparse, and, therefore, other platforms had to be elucidated.

It is due to the aforementioned limitations that, over the years, various devices have been developed in an attempt to reduce the impact of gravity and simulate a near-weightlessness environment (s-*μg*) on Earth. From a physical point of view, gravity is a force exhibiting both magnitude and direction. Therefore, the influence of gravity can be reduced by either manipulating magnitude or direction. An orbital space flight as on the ISS is physically identical to a free-fall. Here, the gravitation acts in a perpendicular manner on the spacecraft's velocity vector, effectively changing its direction constantly but not affecting its magnitude. Free-fall is also found when using sounding rockets, which provide r-*μg* during a time span of up to 15 minutes. On Earth, r-*μg* can also be attained, although only for periods in the range of seconds, in drop towers, and during parabolic flights missions [49, 50]. Although time periods of seconds or minutes limit their use for tissue engineering studies, such periods can be useful to explore various intra- and intercellular processes, responsible for gene expression and protein content changes which can be observed after only a few hours of culturing cells in *μg* [49–51].

## 3. Devices Simulating Microgravity on Earth

In this respect, we should mention an instrument that was introduced by the European Space Agency (ESA) in the early nineties, called the free fall machine (FFM) [52]. This instrument was specifically developed for biological experiments and could generate a free fall for a period of about 800 ms with an intermediate “bounce” of ~20 g for around 50 ms. The paradigm of the FFM is that cells might not be sensitive to the relatively short period of 50 ms of hypergravity, while they experience the relatively longer period of free-fall. Long-term experiments (hours, days), which might be useful for tissue engineering studies, could be performed on this platform. However, thus far, only two studies were published using the FFM, one investigating* Chlamydomonas *[53] and another one researching T-lymphocytes [54]. The* Chlamydomonas* study showed similar results to what was found in real space flight while the T-lymphocytes experiments did not. Considering the very limited number of studies performed on this ground-based device, the FFM still might deserve some more exploration.

Levitating magnets are also used to produce s-*μg* on Earth. Such systems compensate the magnitude of the gravity vector by preventing sedimentation of relatively heavy structures, like cells, by the application of a high gradient magnetic field. This principle was first described for biological systems by Berry and Geim in 1997 [55], who demonstrated that a toad could be levitated and survive while exposed to a 16 Tesla magnetic field. Various experiments in cell biology have made use of such systems [56–58]. The magnetic field acts on individual molecules and atoms within a cell, based on their magnetic susceptibility, preventing them from sedimentation. However, the magnetic field as such confounds possible s-*μg* effects. The direction of the field might force (bio-)polymers into a certain orientation. Different polymers within a cell or on the cell membrane have different susceptibilities, possibly producing artifacts by forcing polymers into specific arrangements, which may not reflect the actual physiological situation [59–61]. Superconducting high gradient magnets are especially capable of performing long-term experiments and might be useful in the area of tissue engineering [62–64]. In this context, another promising technique should be mentioned. This method is the use of magnetic particles for 3D cell cultures. It is not based on a high-gradient magnetic field, but on ferromagnetic particles attached to cells, which can subsequently be levitated by a conventional magnet facilitating the formation of 3D structures [65, 66].

Another option is to manipulate the direction of the gravity vector with respect to the sample. The reduction of the gravitational impact on biological systems by constantly changing its orientation was shown first in experiments by the German botanist von Sachs in 1879, growing* Lepidium sativum* and* Linum usit *[67]. He constructed a slowly rotating system and named it a clinostat, in which, for example, a plant can be placed horizontally and rotated around its longitudinal axis. In doing so, the gravity vector stimulus is constantly changing its impact angle on the sample. As a result, a plant grows straight without the characteristic gravitropic curvature seen when the plant is placed horizontally and not rotating. Based on these initial studies, other rotating systems like the fast rotating clinostat have been developed.

The initial clinostats were rotating relatively slowly in a range from one rotation per couple of hours up to a maximum of about 10 rpm. This is adequate for relatively “solid samples” such as plants, but too slow for cell culture systems that involve a large liquid phase. In a biphasic system, that is, a liquid with particles (cells) both of different density, the heavy particles tend to settle. Rotating such a system around a horizontal axis keeps the heavy particles in suspension. This phenomenon depends mainly on the relative density of the liquid and the particles, the viscosity of the liquid, the rotation speed, and the diameter of the rotated container. When a cell is in a static vessel and the vessel is rotated by 90°, the cell will settle in the direction of the gravity vector. One can repeat this for a full 360° and upon an increase in the frequency of rotation, the traveling distance of the cell decreases. If this rotation is performed constantly with increased speed, we finally end up rotating a cell around its own axis. Such a controlled rotation not only applies to the cells, but also its surrounding boundary liquid phase [68].

Another well-known device to simulate *μg* is the so-called random positioning machine (RPM), a 3D clinostat [69] consisting of two frames, each driven by a dedicated motor. This allows a randomized movement of both frames, independent of each other [69–74]. One of the advantages of the RPM is its size, as cell culture flasks can easily be mounted on it, so it is possible to work with quite large liquid volumes. This ranges from regular T25 flasks [75, 76] to multi-well plates [77], flasks on slides [78], or more dedicated devices [79]. As cells move freely within the liquid, they usually interact with each other and form multicellular spheroids.

**Figure 1:**
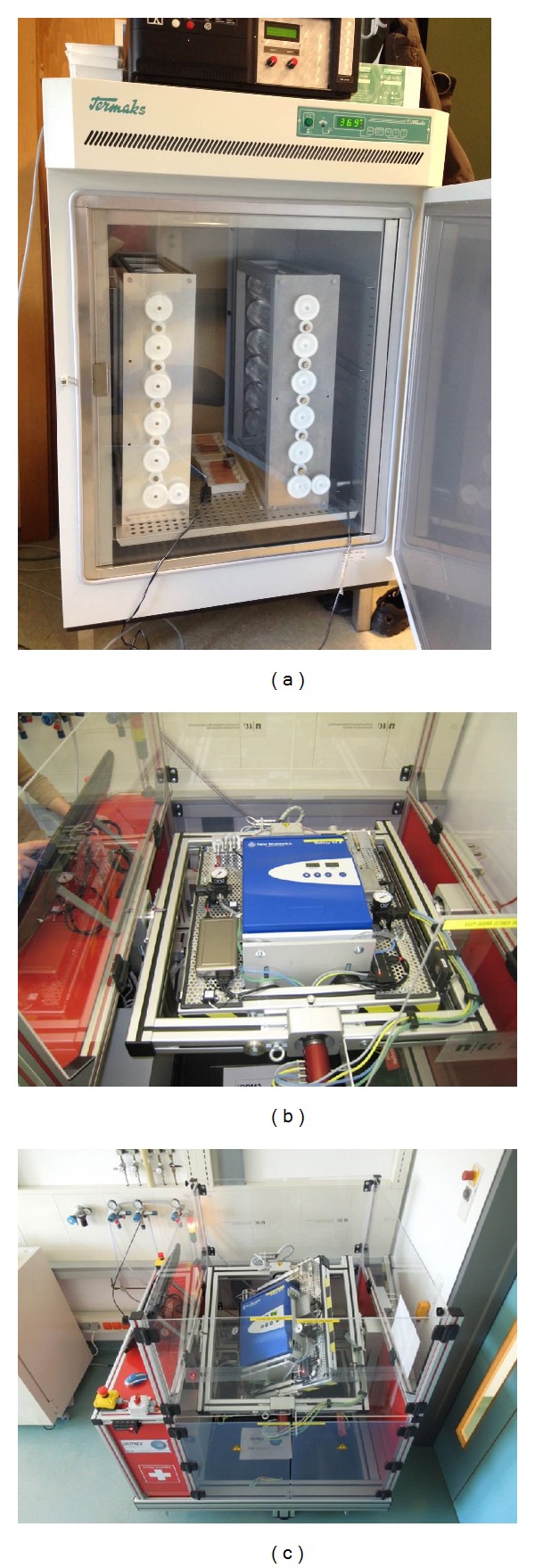
(a) Two 2D clinostat devices in an incubator constructed by the German Aerospace Center (DLR), Institute of Aerospace Medicine, Biomedical Science Support Center, Gravitational Biology, Cologne, Germany. (b, c): Random Positioning Machine simulating microgravity. It was developed by T. Hoson in Japan and manufactured by Dutch Space (former Fokker Space). The basic principle consists of an inner and an outer frame rotating independently from each other in random direction. The samples in the center of the machine experience low gravity as the gravity vector is averaged to zero over time. The redesign of the classical RPM with a CO_2_-Incubator with temperature and CO_2_-level control was realized by Professor Jörg Sekler, Fachhochschule Nordwestschweiz (FHNW), Institut für Automation, Switzerland, and tested by PD Dr. Marcel Egli, Hochschule Luzern—Technik & Architektur, CC Aerospace Biomedical Science & Technology, Hergiswil, Switzerland.

The best simulation of *μg* is achieved in the rotation center of the two axes, which limits the preferred volume size of the samples. Depending on the speed of rotation and the distance from the center, an acceptable residual gravity can be obtained in the order of 10^−4 ^g by a maximum angular velocity of 60° s^−1^ at a radial distance of 10 cm [70]. Earlier RPM models had no possibility to add constituents during the experiment, but newer models have been developed to enable fluid management during rotation [73, 74]. RPMs are commercially available by Mitsubishi Heavy Industries (Kobe, Japan) and Dutch Space (Leiden, The Netherlands), while various academic groups developed similar systems on their own [80–84] (Figure 1).

The rotating wall vessel (RWV) prevents cells from settling via a constant rotation. It has been developed by NASA [85] and is now commercially available through Synthecon Inc. (Houston, TX, USA). Basically, RWVs consist of a slow rotating, relatively large liquid filled container (vessel). The rotation speed has to be adapted to the specific weight of the cells, the fluid density, and viscosity. The cells and tissues in the RWV are constantly falling within the fluid. The settling velocity and direction combined with the rotation of the fluid create spiral trajectories within the vessel [86]. This motion of the sample relative to the fluid generates fluid shear forces on a particle surface ranging from 180 to 320 mPa (1.8–3.2 dyne/cm^2^) for 50 *μ*m beads [87], ~500 mPa (5 dyne/cm^2^) with 3D aggregates of BHK-21 cells [88] to 520–780 mPa (5.2–7.8 dynes/cm^2^) for a 200 or 300 *μ*m spherical object [89]. Over the years, various models based on the initial RWV have been developed, differing in vessel geometry, aspect ratio, and gas supply, such as the slow turning lateral vessel (STLV) [90], the high aspect ratio vessel (HARV) [91], or the rotating-wall perfused vessel (RWPV) [92].

Hence, it can be concluded that annulling the gravity forces, which pull the cells constantly towards the Earth, deliver the ultimate trigger to eukaryotic cells to leave a cell monolayer and assemble in 3D aggregates [5].

It is still unknown which cellular and biochemical mechanisms are involved in the altered signal transduction and in the change of the cellular growth behavior.

## 4. Transition from Two- to Three-Dimensional Cell Growth

A few publications appeared in the literature in recent years, providing some clues for understanding the weightlessness-induced transition from two- (2D) to three-dimensional (3D) cell growth.

Several signaling pathways are affected by annulling gravity forces in the cell interior [93]. However, it is unknown which of these signaling pathways contribute to the formation of three-dimensional aggregates. When endothelial cells form tubes, the nitric oxide signaling pathway appears to be affected [94]. Siamwala et al. reported that iNOS (inducible nitric oxide synthase) acts as a molecular switch, which controls whether the effects of *μg* on vascular endothelial cells induce angiogenesis via the cyclic guanosine monophosphate (cGMP)-PKG-dependent pathway [94]. iNOS is upregulated in HUVEC by a mechanism dependent on suppression of AP-1, after clinorotation of the cells [95]. In addition, the endothelial nitric oxide synthase is phosphorylated by phosphoinositide 3-kinase under weightlessness, simultaneously with Akt [96]. The organoid formation by PC12 pheochromocytoma cells in a RWV bioreactor is accompanied by prolonged activation of the ERK, p38, and jnk signaling pathways [97].

3D cell culture techniques have attracted much attention, not only among biologists, but also clinicians interested in tissue engineering [98, 99] of artificial vessels [100–104] or cartilage [105–108]. Moreover, osteoarthritis and cartilage trauma occur in patients with a high incidence, but current treatment methods are still limited [109]. Even a minor injury to articular cartilage may lead to progressive damage and degeneration [110].

## 5. Tissue Engineering of Bone 

Bone loss has been documented for many years in *μg* (1-2% a month). Increased bone loss and risk of fractures is an identified risk in the bioastronautics critical roadmap for long-term cosmic missions to the moon and mars.* In vitro* drug screening both in 1 g, *μg* and in artificial gravity is essential to adequately address countermeasures for bone loss. Bone loss in *μg* is the second most important risk to space missions [5, 6].

Exposure to the *μg* environment of space causes astronauts to lose calcium from bones [5, 6]. This loss occurs because the absence of Earth's gravity disrupts the process of bone maintenance in its major function of supporting body weight. Exposure to the *μg* environment of space causes men and women of all ages to lose up to 1% of their bone mass per month due to disuse atrophy, a condition similar to osteoporosis. It is not yet clear whether loss in bone mass will continue as long as a person remains in the *μg* environment or level off in time.

There are, indeed, four major bone cell types, and each of them seems to be influenced by *μg*. Bone mesenchymal stem cells (MSC) are able to differentiate into adipocytes, osteoblasts, and osteoclasts. Proliferation and differentiation are very sensitive to *μg*, as the lack of gravity in space can reduce mechanical stress, leading to a decreased rate of osteogenesis and an increased adipogenesis rate [111]. As the signaling pathways involved in MSC differentiation form a complicated network, it has been found that the reduction in the osteogenesis of MSCs in the presence of *μg* is mediated by a decrease in the integrin/mitogen-activated protein kinase (MAPK) signaling pathway [112], as well as RhoA and cytoskeletal disruption [113].

Osteoblasts are derived from MSCs, but in *μg* the differentiation does not function properly, and the resulting bone loss has been attributed to osteoblasts due to their (1) reduced proliferation and activity, (2) reduced differentiation, and (3) decreased responsiveness to bone-related factors in the microenvironment [114]. Observations have also been made regarding the cytoskeleton of osteoblasts; there is growing evidence that the cytoskeleton is closely connected to nuclear morphology and function [115]. The enlarged nuclei observed in flight osteoblasts could be a result of cytoskeletal disruption [116].

Osteocytes regulate bone resorption and formation and are considered the terminal differentiation stage of osteoblasts. The osteocytes in cortical bone and periosteum degenerated after a 12.5-day flight in space on the Cosmos Biosatellite [117]. Osteocyte apoptosis has been observed after a 2-week flight, increasing the number of functionally active osteoclasts [118]. Apoptotic osteocytes are essential for the initiation of bone remodeling, but it is the neighboring nonapoptotic osteocytes that produce proosteoclastogenic signaling [119]. Osteocytes seem to be the key effectors of *μg* induced bone loss [120].

Osteoclasts are bone-resorbing cells, and their differentiation seems to be enhanced in *μg* [121]. This could be another explanation of bone-loss in space.

The mystery, for the moment, is what signals permit bone tissue to adapt to a weightless or an Earth (1 g) environment. Researchers do not yet know whether the biomechanical stimuli that are changed by *μg* directly affect osteoblast and osteoclast function or if other physiological factors such as hormone levels or poor nutrition contribute to bone loss. NASA investigators are studying gravity-sensing systems in individual bone cells by flying cultures of these cells on the space shuttle and observing how they function. Discoveries made in the course of space biomedical research on bone are already contributing to a better understanding of osteoporosis and the treatment of bone mass loss on Earth as well as in space. The single most important contribution that NASA research has made to the understanding of bone deterioration in osteoporosis is heightened awareness of the importance of gravity, activity, and biomechanics—that is, the mechanical basis of biological activity— in bone remodeling.

Mechanical forces—the action of energy on matter—appear to coordinate bone shaping processes. The standard theory of bone remodeling states the body translates mechanical force into biochemical signals that drive the basic processes of bone formation and resorption. Aging, especially in postmenopausal women, and exposure to *μg* uncouple bone resorption and formation. When this uncoupling occurs, formation lags behind resorption, and the result is bone loss.

Researchers are not yet certain whether bone resorption speeds up or the bone formation slows down, though recent experimentation in space indicates that *μg* might somehow affect both processes. Progress in developing methods of preventing or treating disuse atrophy and osteoporosis depends on better understanding of the mechanisms that cause the problem. Determining how the body translates mechanical loading (physical stress or force) into the signals that control bone structure may reveal how aging, inactivity, and space flight uncouple bone formation and resorption. Only in the absence of gravity can we determine the influence of weight and stress on bone dynamics.

By studying what mechanisms translate mechanical stress on bones into biochemical signals that stimulate bone formation and resorption, space life scientists may be able to determine how to maintain bone mass. Researchers do not yet know exactly what type and amount of exercise, hormones, or drugs might prevent bone loss or promote bone formation. However, some combination of sex hormones, growth hormones, and exercise seems to be the key to preventing bone mass loss associated with chronological aging and postmenopausal hormone changes on Earth.

**Figure 2:**
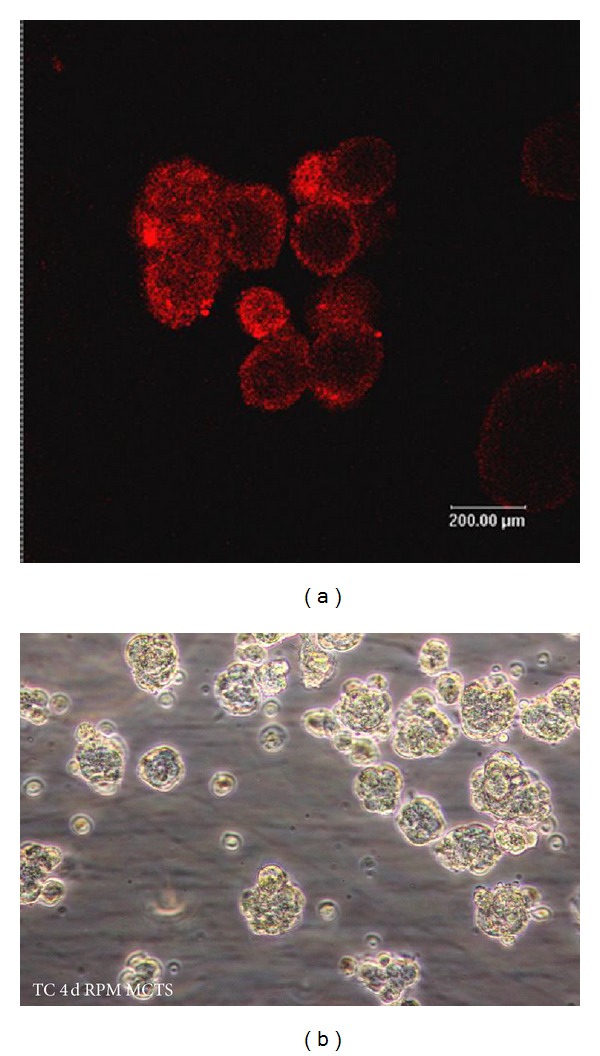
(a) Production of large numbers of small (200 *μ*m diameter) immature (7-day-old) osteospheres with labeled osteoclast cells (red) viewed by confocal imaging in living constructs-USPTO 80736136 and (b) follicular thyroid cancer cells (TC) cultured on the RPM. Several multicellular tumor spheroids are visible after 4 days.

Bone is made up of several different cell populations. Osteoclasts are responsible for the breakdown of mineralized bone, in preparation for bone remodeling. In contrast, the osteoblasts synthesize mineralized bone in the remodeling process. The goal of this project is to develop an “*in vitro*” three-dimensional, cellular model of osteoclasts and osteoblasts (human and rodent) cultured together in *μg* analog culture conditions to identify the underlying biomarkers related to bone loss in *μg* and the cellular mechanisms involved in bone resorption. The NASA rotating-wall vessel (RWV) permits the growth of mixed cell cultures for much longer periods than traditional culture methods. This would set the stage for development of countermeasure strategies for bone loss in space as well as in osteoporosis and rheumatoid arthritis which are increased health risks on Earth. Professor Sundaresan and collaborators [122–124] have developed a 3D cell culture bone tissue model using a specialized rotating-wall vessel culture system to address a more physiologically relevant model to the human body. The use of the cells by themselves also eliminates confounding variables such as neuroendocrine stress found* in vivo* (Figure 2(a)).

The human body needs a framework to withstand gravity. This framework is given by the skeletal system. During long-term space missions, bone loss has been reported in astronauts at a rate that is both substantial and progressive with time spent in *μg* [125–128]. But what is the reason for this massive bone loss? Some studies suggested that this effect might be attributed to increased resorption in load-bearing regions of the skeleton [129–131], and evidence of a decrease in bone formation had also been described. For example, the loss of bone in *μg* is about 10 times greater than the bone mineral density loss per month of postmenopausal women on Earth, who are not on estrogen therapy [132–135]. The loss of bone mineral density in a six-month mission appeared to be reversible in 1000 days after return to Earth [136, 137], but changes in the bone structure are irreversible and seem to mimic changes in the elderly [137].

Until now there are still knowledge gaps on the mechanism of bone loss, especially on the molecular and cellular mechanisms, also the question of fracture repair arises. Moreover, more information is needed on the influence of radiation, hormones, and fluid shifts.

Investigations in humans and animals are quite difficult due to the lack of long-term flight opportunities, the absence of animal housing facilities in space, and the problem of material collection from returning astronauts. Thus, other possibilities have to be sought in order to investigate bone. So far, most commonly used are bone cell culture experiments, which are a viable opportunity for investigating cells in 3D, acting as tissue like samples while they are cultivated under conditions of weightlessness. However, 3D embryonic bone tissue cultures have been used in the past and show a clear decrease in matrix mineralization, in mineralizing cartilage and by osteoblasts, combined with an increased mineral resorption by osteoclasts [138].

Besides this, tissue engineering is a very up-to-date topic. The ultimate goal is to generate functional 3D constructs, which can be used as replacement organs or structures with normal function or serve for* in vitro* studies [5, 139]. Bone replacement, especially, is quite difficult, as large bone defects usually require reconstructive surgery to restore function [140]. Up to date, the treatment includes autograft or allograft transplantation and the use of synthetic materials [141]. While autograft transplantation is the preferred treatment, it suffers from limited supply and donor site morbidity [142]. As the autogenous origin of cells prevents potential immune rejection, the amount of bone marrow suitable for transplantation is limited. New techniques have been developed, allowing selection of bone marrow osteoprogenitor cells and expanding them in culture, so that a large amount of transplantable cells can be generated after only one biopsy [143–145].

In principle, culturing bone cells is not that easy. A combination of osteoconductive matrices, bone-forming cells, and osteogenic growth factors is needed for the engineering of bone tissue [146]. The first important factor is the cell type. Osteoblasts are in a close to mature stage, showing a low proliferative potential. Mesenchymal stromal cells represent a proliferating and undifferentiated cell source, but their availability is limited [147, 148]. An option to increase their lifespan* in vitro* is the overexpression of human telomerase reverse transcriptase (hTERT). The second factor is an ideal scaffold, which possesses mechanical properties comparable to bone. It should support cell adhesion and should be biodegradable to facilitate natural bone remodeling [146]. As of now, different studies have shown the advantages and disadvantages of several types of scaffolds like chitin, gelatin, poly(lactic acid), poly(glycolic acid), poly(lactic acid-co-glycolic acid), polycaprolactone, hydroxyapatite, coral, and so forth. Several* in vitro* studies revealed an ideal scaffold pore size for osteoblasts from 200 to 400 *μ*m [149, 150]. It is important to recognize that the scaffold architecture influences the distribution of shear stress, the range of mechanical stimuli, as well as the proliferation and differentiation of osteoprogenitor cells [151, 152].

To simulate an ideal* in vivo* situation for* in vitro* cells, specific cytokines and growth factors are necessary. For bone morphogenesis, the bone morphogenetic proteins (BMP), which belong to the transforming growth factor beta (TGF-*β*) superfamily, are essential [153]. Currently, only BMP-2 and -7 are commercially available, so alternatives to stimulate osteoprogenitor cells by growth factors are required. It has been reported that autologous platelet-rich plasma is an effective bioactive supplement, as it contains osteogenic and angiogenic growth factors [154].

Several different bioreactor systems are already available for bone tissue engineering. A well-known and simple system is the spinner flask bioreactor. Convective forces are provided by a stirrer and the medium flows around the cells. The emerging shear stress is not applied homogenously, as there appears to form a gradient in the flask [146]. This factor certainly needs to be considered when conducting studies with the spinner flask system.

Other suitable instruments are rotating bioreactor systems, for example, the RWV. It has been used with different kind of bone cells, which are often grown with the help of microcarriers [8, 155] or scaffolds [8–11, 15, 155]. The high aspect ratio vessel (HARV) [91] was used by Lv et al. [12] to engineer tissue on poly(lactic acid glycolic acid)/nano-hydroxyapatite composite microsphere-based scaffolds.

Some researchers used bone marrow mesenchymal stem cells for their investigations. Jin et al. [16] were able to transplant RWV-grown bone constructs in cranial bone defects of Sprague-Dawley rats and found them to be more effective in repairing the defects than the 1 g controls after 24 weeks. Moreover, a 3D environment as in a rotary cell culture system enhanced osteoblast cell aggregation and mineralization [13]. Preosteoblasts cultured in a RWV could be engineered into osseous-like tissue [14].

## 6. Mesenchymal Stem Cells and Microgravity

Mesenchymal stem cells (MSCs) are cells capable of long-term proliferation and differentiation into various stromal tissue cell types. The state of MSCs rests on the cellular microenvironment and several soluble factors. In addition, gravity can influence MSC features. Disuse, as encountered during long-term bed-rest or space travel, and the accompanying absence of mechanical stimuli lead to an inhibition of osteogenesis and simultaneously to an induction of adipogenesis in MSCs. Hence, it is crucial to provide a proper mechanical stimulation for cellular viability and osteogenesis, particularly under unusual conditions.

In 2004, Merzlikina et al. [27] studied the effects of prolonged clinorotation on cultured human MSC morphology, proliferation rate, and expression of specific cellular markers. After exposure of the cells to clinorotation for time frames from 1 h to 10 days, it was shown that the proliferative rate decreased in the experimental cultures as compared to cells growing under normal conditions. Clinorotated MSCs seemed more flattened and reached confluence at a lower cell density, which advocates that cultured hMSCs sense the changes in the gravity vector and respond to s-*μg* by altered functional activity. The group around Myoui [28] examined whether gravity-induced stress is linked to osteoblast differentiation and function. Rat marrow mesenchymal cells (MMCs) were cultured in pores of interconnected porous calcium hydroxyapatite (IP-CHA) for 2 weeks on a 3D clinostat. In MMCs subjected to s-*μg*, the marker of osteoblastic differentiation alkaline phosphatase activity was decreased by 40%, compared to the control group. Also, the clinostat group exhibited less extensive extracellular matrix formation than the control group. The implantation of the IP-CHA/MMC composites in syngeneic rats showed that bone formation was significantly lower for the clinostat group than for the control group. Yuge et al. [29] also used a 3D clinostat for their experiments on the proliferation behavior of hMSCs. The proliferation rate of the cells of the clinostat group was elevated almost 3-fold in comparison to the control group, and the number of hMSCs double-positive for CD44/CD29 or CD90/CD29 in the clinostat group after 7 days in culture increased 6-fold. The hMSCs cultured in a 3D-clinostat were still able to differentiate into hyaline cartilage after transplantation into cartilage defective mice and displayed the strong proliferative characteristic of stem cells, thus, showing that s-*μg* may be used to expand stem cell populations* in vitro*. In contrast to these findings, Dai et al. [24] reported in 2007 that *μg* simulated by a clinostat inhibited population growth of bone marrow mesenchymal stem cells (rBMSCs) and their differentiation towards osteoblasts. The cells grown on the clinostat were arrested in the G(0)/G(1) phase of cell cycle, and growth factors, such as insulin-like growth factor-I, epidermal growth factor, and basic fibroblast growth factor had only a slight stimulatory effect compared to the static control group. Gershovich and Buravkova's [17] work supports this hypothesis. After 20 days of clinostat-exposure, the proliferative activity of hBMCs was reduced, whereas it increased the number of large flat cells in the culture and stimulated migration activity of cells. In 2009, Gershovich and Buravkova [30] demonstrated the effects of s-*μg* by clinostat and RPM on the interleukin production by hBMSCs and MSC osteogenous derivatives. 20-day exposure on a clinostat increased the interleukin-8 (IL-8) content 1.4 to 3.2 times in the culture medium, while the average increase of IL-production on the RPM amounted to 1.5–6 times (10 days) and 1.6–2.1 times (20 days), respectively. This suggests that results of s-*μg* vary by the use of different modeling systems. rMSCs grown in a clinostat demonstrate that s-*μg* can boost the differentiation of MSCs into neurons, as demonstrated by Chen et al. [156] In s-*μg*, neuronal cells derived from rMSCs were found to express higher microtubule-associated protein-2 (MAP-2), tyrosine hydroxylase (TH) and choline acetyltransferase (CHAT). Furthermore, the excretion of neurotrophins such as nerve growth factor (NGF), brain derived neurotrophic factor (BDNF), or ciliary neurotrophic factor (CNTF) was increased. In comparison to 1 g controls, neuronal cells from the s-*μg* group generated more mature action potentials and displayed repetitive action potentials. This might benefit the search for new strategies for the treatment of central nervous system diseases.

Zayzafoon et al. [18] demonstrated that s-*μg* inhibits the osteoblastic differentiation of hMSC and induces the development of an adipocytic phenotype. In the effort of understanding space flight-induced bone loss, the group used the rotary cell culture system (RCCS) to model *μg* and determine its effects on osteoblastogenesis. Human MSCs were cultured and osteogenic differentiation was induced before the initiation of s-*μg*. As a result, the important mediator of adipocyte differentiation, peroxisome proliferator-activated receptor gamma (PPARgamma2), and adipsin, leptin, and glucose transporter-4 was highly expressed. These changes were not adjusted after 35 days of readaptation to normal gravity. Moreover, *μg* decreased ERK- and increased p38-phosphorylation pathways, known to regulate the activity of runt-related transcription factor 2 and PPARgamma2. These results were supported by Saxena et al. [19] in 2007, who demonstrated that s-*μg* inhibited osteoblastogenesis and increased adipocyte differentiation in hMSCs incubated under osteogenic conditions using the RCCS. They could show that a reduced RhoA activity and cofilin phosphorylation, disruption of F-actin stress fibers, and decreased integrin signaling through focal adhesion kinase were involved in this process. Meyers et al. [20] also investigated the effects of s-*μg* on integrin expression and function in hMSCs, since a reduced osteoblastic differentiation might be caused by impaired type I collagen (Col I)-integrin interactions or a reduction of integrin signaling. Culturing of hMSCs for 7 days in s-*μg*, lead to reduced expression of Col I, while Col I-specific alpha2 and beta1 integrin protein expression increased. However, autophosphorylation of adhesion-dependent kinases, focal adhesion kinase (FAK) and proline-rich tyrosine kinase 2 (PYK2) was significantly reduced. These findings indicate that a reduction in osteoblastogenesis in s-*μg* is, at least in part, caused by a reduced integrin/MAPK signaling. The group around Duan [16] studied the relationships between the composition and mechanical properties of engineered bone constructs. BMSCs were grown for 15 days on ceramic bovine bone scaffolds in different environments, namely, static flasks and the RWV. DNA content and alkaline phosphatase (ALP) were higher for cells grown on the RWV. After transplantation into Sprague-Dawley rats with cranial bone defects, the bone constructs engineered on the RWV repaired the defects better and showed histologically better bone connection.

Sheyn et al. [21] evaluated the effect of s-*μg* on all genes expressed in hMSCs, with the hypothesis that many important pathways are affected during culture on a rotating wall vessel system. The analysis of gene expression by use of whole genome microarray and clustering showed that 882 genes were downregulated and 505 genes were upregulated after exposure to s-v. A multitude of genes belonging to cell compartment, biological process, and signaling pathway clusters were modulated, as identified by gene ontology clustering. Significant reductions in osteogenic and chondrogenic gene expression and an increase in adipogenic gene expression were shown and could be validated by a parallel adipogenic differentiation assay. In order to investigate the effects of s-*μg* on chondrogenic differentiation of human adipose-derived MSCs (ADSCs), Yu et al. [22] cultured cells on a RCCS in pellets with or without the chondrogenic growth factor TGF-*β*_1_. Analysis of real-time PCR and histological results demonstrated that s-*μg* has a synergistic effect on chondrogenesis with TGF-*β*_1_. The p38 MAPK pathway was activated by TGF-*β*_1_ alone and was additionally stimulated by s-*μg*. Inhibition of p38 activity with SB203580 suppressed chondrocyte-specific gene expression and matrix production. This indicates that the p38 MAPK signal mediates s-*μg*-induced chondrogenesis of ADSCs. In MSCs cultured during chondrogenic induction in a rotating culture, combined with polyglycolic acid (PGA), mRNA and proteins of collagen type II and aggrecan were significantly more expressed in the s-*μg* rotating culture group than the static culture group, as reported by Wu et al. [25]. Zhang et al. [26] described that MSCs spread out in a spindle shape when cultured in normal gravity, while they become unspread and round under s-*μg*. Also, under s-*μg*, their cytoskeleton fibers are being reorganized. The function of MSCs was affected by these morphological changes, transmitted through the activity of RhoA. To test the hypothesis that s-*μg* has the capacity to offer a novel choice in the stimulation of neovascularization, MSCs were cultured under s-*μg* stimulation followed by VEGF differentiation. The responses revealed that MSCs were differentiated into endothelial-like cells after 72 h incubation and were able to form a capillary network. Their endothelial differentiation potential improved compared with the static control group.

Another approach of modeling *μg* in hMSCs is the use of a large gradient high magnetic field (LGHMF) produced by a superconducting magnet. Shi et al. [64] analyzed the effects of LGHMF-*μg* on survival, cytoskeleton and osteogenic potential of hMSCs. Results showed that the LGHMF-*μg* treatment disrupted the cytoskeleton of hMSCs, a LGHMF-*μg* treatment for 24 h led to cell death. LGHMF-*μg* treatments in early stages of osteogenic induction resulted in suppression of osteogenesis of hMSCs. The suppression intensity was reduced gradually as the treatment stage of LGHMF-MG was postponed. A LGHMF-*μg* treatment during the ending-stage of osteogenic induction had no visual effect on osteogenesis of hMSCs, which indicates that LGHMF-*μg* affects the initiation of osteogenesis.

Furthermore, a study of Uddin and Qin [23] examined the effects of low intensity pulsed ultrasound (LIPUS) on the osteogenic differentiation of adipose-derived human stem cells (Ad-hMSC) under s-*μg* conditions. Microgravity was simulated in a 1D clinostat and treated with LIPUS at 30 mW cm^−2^ for 20 min day^−1^. Hypothetically, the application of LIPUS to s-*μg* cultures would restore osteogenesis in Ad-hMSCs. The results showed significant increases in* ALP*,* OSX*,* RANKL*, and* RUNX2* and decreases in* OPG* gene expression in LIPUS treated SMG cultures of Ad-MSC compared to nontreated cultures. LIPUS also restored* OSX*,* RUNX2*, and* RANKL* gene expression in osteoblast cells. s-*μg* significantly reduced ALP positive cells by 70% (*P* < 0.01) and ALP activity by 22% (*P* < 0.01), while LIPUS treatment restored ALP positive cell number and activity to equivalence with normal gravity controls. Extracellular matrix collagen and mineralization was assessed by Sirius red and Alizarin red staining, respectively. s-*μg* cultures showed little or no collagen or mineralization, but LIPUS treatment restored collagen content to 50% (*P* < 0.001) and mineralization by 45% (*P* < 0.001) relative to s-*μg*—only cultures.

## 7. Multicellular Tumor Spheroids

3D growth of tumor cells creating MCTS* in vitro* has been observed in various tumor cell lines including thyroid and colorectal cancer [31]. MCTS mimic the growth of solid tumors and represent a simple model, approaching some of the characteristics found* in vivo* including physiological characteristics such as multicellular architecture and natural barriers of mass transportation. Therefore, the use of MCTS as an* in vitro *tool for testing anticancer drugs has gained significant interest as MCTS potentially provide a more reliable model for drug testing compared to single layer adherent cell cultures. During the approval process of drugs before clinical testing in trials, the mechanisms of delivery and the effectiveness of the drugs must be determined. The first steps of preclinical drug testing are typically carried out using adherent cell formats growing in two dimensions [157]. However, the outcome of such investigations in two-dimensional cell systems is often very different from what is observed in a whole-body situation. This makes it difficult to draw clear conclusions of the drug properties anticipated* in vivo*. In terms of drug delivery, a spheroid test platform has inherent advantages, providing a natural barrier resembling the natural tumor environment. Spheroids of a particular size exhibit certain gradients of oxygen and nutrition [35–37, 158, 159]. Spheroids larger than 400–500 *μ*m in diameter show characteristics of layered structures with a hypoxic core, consisting of necrotic cells surrounded by quiescent cells and an outer layer of proliferating cells [38, 160, 161]. Hence, 3D tumor cell systems are a valuable tool for studying drug delivery and the response and metabolism of hypoxic tumor cells to cancer therapy. Fang et al. reported that multicellular spheroids of primary human colon cancer cells were resistant to chemotherapy-induced apoptosis and retained the expression of colon cancer marker CD133, mimicking colorectal cancer [162]. Were these cells grown under normal conditions, they did not retain these characteristics. Size control of MCTS is a major challenge in obtaining uniform and reliable high throughput test systems; various techniques such as forced aggregation techniques, micro textured surfaces, and porous 3D scaffolds are being employed to solve these issues [163–166]. There are several conventional methods for generating 3D aggregates of cancer cells, including NASA rotary cell culture systems, hanging drops, and culturing of cells using nonadherent surfaces [166–168]. Conditions of s-*μg* using the RPM (Figure 2(b)) or the HARV have been shown to induce the growth of MCTS without the use of scaffolds in several types of human cancer cells [31–33, 39, 169]. The molecular switches initiating s-*μg*-induced spheroid formation are still unknown. Several changes in morphology and gene expression profiles have been observed in follicular thyroid cancer cells, grown under s-*μg* conditions with the main features involving changes in the ECM and early induction of apoptosis [39, 40]. Signaling between exogenous ECM and tumor surface receptors has long been thought to be an essential component in regulating the tumorigenic phenotype in 3D cultures. These phenomena have been demonstrated in studies showing that blocking specific ECM-integrin signaling can cause a shift in the malignant potential of tumor cells, leading to a more benign phenotype [40, 170]. In an attempt to identify gravity sensitive genes responsible for MCTS formation, Grosse et al. [41] identified 487 transcripts, which were differently regulated after 24 h of s-*μg* in comparison to the ground control. Comparing adherent cells with MCTS under s-*μg* conditions revealed significant differences in terms of growth patterns and signaling. Interestingly, the rate of apoptosis was increased in adherent cells compared to MCTS, indicating that the early phase induction of apoptosis may be concomitant with the transition of cells shifting from 2D to 3D growth. Several NF-*κ*B-driven genes, involved in the regulation of tumor invasion, were upregulated by s-*μg* in adherent cells, highlighting that s-*μg* initiates distinct adaptive mechanisms in the cells.

## 8. Summary 

The development of tools like the RPM or RWV gave new impacts in the field of tissue engineering (Table 1). Growing cells in simulated or real weightlessness, for example, on the RPM, might be a highly promising new technique to generate tissue constructs in a scaffold-free manner. Cultivation of chondrocytes might lead to small cartilage particles, which could be used to replace injured or outworn cartilage. Restoring normal osteogenic differentiation of MSCs from s-*μg* exposure by daily short-term stimulation could be helpful so that tissue products may become commercially available, like it has already happened for some bone tissues (BioSeed-Oral Bone, co.don osteotransplant and Osteocel).

At the moment, studies analyzing the molecular mechanisms behind spheroid formation of, for example, thyroid cells, chondrocyte, and others have increased the knowledge of the complex regulation of 3D growth in *μg* [42–45, 171].

To be able to use this new technique more efficiently, further studies are necessary to better understand the exact cellular changes specific to these conditions. Tissue, which was produced under s- or r-*μg* conditions, might be helpful to better understand cell signaling, intercellular contact, and tissue growth as well as being sufficient for medical transplantation. MCTS can be used as an alternative to animal experiments.

Although the *μg* environment is not a common field for biologists and medical researchers, recent studies have clearly shown that the loss of gravity impacts the cells and it dramatically changes the genome, proteome, and secretome of these cells [43, 45]. Therefore, it is important to systematically explore the advantages of this new research opportunity. Different space flights have already demonstrated a 3D cell growth (Table 2) and similar results have been detected with the help of devices simulating *μg* in ground-based laboratories [34, 44, 46, 172–174].

## Figures and Tables

**Table 1:** Comparative methods of 3D cell culture systems using simulated *μg*.

Device		Working principle
Free fall machine	FFM	Free fall for 800 ms, “bounce” of 20 g for 50 ms

Levitating magnets	LM	A high gradient magnetic field prevents sedimentation

2D-clinostat		Rotation along one axis

Random positioning machine	RPM	Two frames with randomized movement

Rotating wall vessel	RWV	Constant rotation prevents cells from settling

**Table 2:** Overview of various cell types used for tissue engineering and *μg*-conditions involved.

Cell type	Engineered tissue	Method	References
Osteoblasts	Bone	RWV bioreactor (s-*μg*)	[8–12]
Osteoprogenitor cells	Bone	RWV bioreactor (s-*μg*)	[13, 14]
Mesenchymal stem cells	Bone	RWV bioreactor (s-*μg*)	[12, 15–23]
Mesenchymal stem cells	Divers	RWV bioreactor (s-*μg*)	[18, 21, 24–26]
Mesenchymal stem cells	Divers	RPM (s-*μg*)	[27–30]
Several cell types	MCTS	RWV bioreactor (s-*μg*)	[31–34]
Several cell types	MCTS	Spinner flask (s-*μg*)	[35–38]
Several cell types	MCTS	RPM (s-*μg*)	[39–44]
Several cell types	MCTS	Space (r-*μg*)	[45]
Hepatocytes	liver	RWV bioreactor (s-*μg*)	[34, 46]
